# Promoter Region Hypermethylation and mRNA Expression of *MGMT* and *p16* Genes in Tissue and Blood Samples of Human Premalignant Oral Lesions and Oral Squamous Cell Carcinoma

**DOI:** 10.1155/2014/248419

**Published:** 2014-06-02

**Authors:** Vikram Bhatia, Madhu Mati Goel, Annu Makker, Shikha Tewari, Alka Yadu, Priyanka Shilpi, Sandeep Kumar, S. P. Agarwal, Sudhir K. Goel

**Affiliations:** ^1^Department of Pathology, King George's Medical University, Lucknow 226003, India; ^2^Petroleum Toxicology Division, CSIR-Indian Institute of Toxicology Research (CSIR-IITR), Lucknow 226003, India; ^3^Department of Biochemistry, Saraswati Dental College, Lucknow 227105, India; ^4^All India Institute of Medical Sciences, Bhopal, Madhya Pradesh 462026, India; ^5^Department of Otorhinolaryngology, King George's Medical University, Lucknow 226003, India; ^6^Department of Biochemistry, All India Institute of Medical Sciences, Bhopal, Madhya Pradesh 462026, India

## Abstract

Promoter methylation and relative gene expression of O^6^-methyguanine-DNA-methyltransferase (*MGMT*) and *p16* genes were examined in tissue and blood samples of patients with premalignant oral lesions (PMOLs) and oral squamous cell carcinoma (OSCC). Methylation-specific PCR and reverse transcriptase PCR were performed in 146 tissue and blood samples from controls and patients with PMOLs and OSCC. In PMOL group, significant promoter methylation of *MGMT* and *p16* genes was observed in 59% (*P* = 0.0010) and 57% (*P* = 0.0016) of tissue samples, respectively, and 39% (*P* = 0.0135) and 33% (*P* = 0.0074) of blood samples, respectively. Promoter methylation of both genes was more frequent in patients with OSCC, that is, 76% (*P* = 0.0001) and 82% (*P* = 0.0001) in tissue and 57% (*P* = 0.0002) and 70% (*P* = 0.0001) in blood, respectively. Significant downregulation of *MGMT* and *p16* mRNA expression was observed in both tissue and blood samples from patients with PMOLs and OSCC. Hypermethylation-induced transcriptional silencing of *MGMT* and *p16* genes in both precancer and cancer suggests important role of these changes in progression of premalignant state to malignancy. Results support use of blood as potential surrogate to tissue samples for screening or diagnosing PMOLs and early OSCC.

## 1. Introduction

Worldwide, oral squamous cell carcinoma ranks the sixth most common cancer and accounts for 3–5% of all human malignancies [1, 2]. It is the most frequent cancer of head and neck region which encompass tumors arising from the epithelium of the nasal, oral cavity, pharynx, and larynx [3]. In India, oral cancer ranks as number one amongst all cancers in males and number three amongst all cancers in females. Tobacco smoking and alcohol consumption are established risk factors associated with OSCC and the most common oral premalignancies [4]. It is reported that different carcinogens activate or inhibit specific pathways during cancer development and progression. The oral habits of tobacco and betel quid chewing, bidi (tobacco flakes wrapped in a tendu leaf) smoking, and alcohol consumption have been documented as risk factors for OSCC in Indian population and the most prevalent tumor sites are mouth and oropharynx [5]. All the above forms of tobacco are known to contain hydrocarbons and several potent nitrosamines which are carcinogenic and act via alterations in DNA thus playing a key role in initiation and promotion of oral cancer [6].

OSCC arises as a result of multiple molecular events that develop from the combined influences of an individual's genetic predisposition, immunodeficiency, and external agents such as dietary factors and viruses and like human papilloma virus (HPV) and Epstein-Barr virus (EBV) [7, 8]. Accumulation of genetic alterations can lead to the development of premalignant lesions and subsequent invasive carcinoma. Correct diagnosis of premalignant oral lesions enables treatment before progression to an invasive neoplasm. The most common premalignant lesions in the oral cavity are leukoplakia with and without dysplasia, submucosal fibrosis (SMF), and oral lichen planus (OLP) [9]. Estimates of the global prevalence of oral potentially malignant disorders range from 1 to 5% [10]. Both genetic and epigenetic changes are known to contribute to tumorigenesis in humans. While genetic alterations refer to irreversible changes in DNA sequence leading to oncogene activation or tumor suppressor gene inactivation [1, 11], epigenetic changes denote reversible and heritable modifications in gene expression without any alterations in the DNA sequence. DNA methylation is the most significant epigenetic modification in eukaryotic genome which plays a key role in many existing cellular pathways including apoptosis, cell adherence, DNA repair, and cell-cycle control and during the development of cancer [12–14]. Aberrant DNA methylation includes genome-wide hypomethylation as well as promoter CpG island hypermethylation [15]. Several studies have reported certain cancer-related genes to be frequently methylated in oral malignancies. Among these are O^6^-methyguanine-DNA-methyltransferase (*MGMT*) and* p16* genes.* MGMT* (located on chromosome 10q26) is a detoxifying agent of DNA adducts and prevents alkylation [14]. It encodes DNA-repair protein O^6^-alkylguanine DNA alkyltransferase (AGT) which repairs potentially mutagenic and cytotoxic alkylation of DNA by removing adducts from the O^6^ position on guanine [16]. It is now well established that DNA adducts are formed by alkylating intermediates under exposure to tobacco intake [17]. Inactivation of* MGMT* occurs rarely owing to deletion, mutation, or rearrangement of the* MGMT* gene [13]. Previous studies have found that loss of* MGMT* protein expression is predominantly associated with hypermethylation of the promoter region in a variety of primary human cancers [14, 16, 18–20] suggesting that inactivation of this DNA repair mechanism may be an important step in human tumorigenesis. Reports on transcriptional silencing of* MGMT* gene in OSCC have shown that frequency of* MGMT* promoter methylation ranges from 23 to 56% in tumor tissue as compared to 9% in healthy oral mucosa suggesting that inactivation of this DNA repair mechanism may be a significant event in oral carcinogenesis [20, 21]. However, loss of expression of* MGMT* by promoter hypermethylation in premalignant oral lesions has received relatively little attention.

The* p16* gene (located on chromosome 9p21) plays a key role in cell cycle regulation and codes for a protein which binds and inhibits cyclin-dependent kinases (CdK-4 and -6) and phosphorylates serine and threonine residues of retinoblastoma (Rb) protein [22, 23]. The* p16* promoter region methylation has been reported to vary between 17 and 43% in oral cancer cell lines, 23 and 67% in primary tumors, and 0% in healthy oral mucosa [24–27]. Frequent* p16* hypermethylation has been observed in premalignant oral lesions by Hall et al. [28] and it was reported that these lesions tend to be transformed into OSCC.

Blood is one of the most easily available (noninvasive) clinical samples and tumor DNA is known to be present in blood stream as cell free DNA circulating in the plasma or serum of cancer patients [29–32]. Genetic and epigenetic alterations including point mutations, microsatellite instabilities (MI), losses of heterozygosity (LOH), and DNA hypermethylations have been reported in circulating DNA. In many cases, these changes were found to be identical to those in the primary tumor tissue of the patient, supporting the tumoral origin of altered cell free DNA [30]. Although presence of alterations in cell free DNA, as well as its overall increase, is not restricted to any particular tumor site, type, or grade, there is however a tendency for significantly larger amounts of circulating DNA in patients with late stage disease and metastasis [29]. Thus, blood may serve as an important surrogate material for cell free DNA-based molecular analysis in cancer and precancer patients. Elucidation of the pathogenesis and establishment of effective treatment are significant challenges in oral oncology; however, many aspects of oral carcinogenesis are still poorly understood. In order to gain a mechanistic insight into the complex molecular events leading to the progression of premalignant oral lesions to cancer, in the present study we looked for the promoter region methylation status of* MGMT* and* p16* genes in the blood and tissue samples of patients with premalignant oral lesions and OSCC. In addition, relative gene expression assay was conducted to analyse transcriptional dysregulation of these two genes in tissue and blood samples of both premalignant and malignant lesions. The methylation induced gene silencing was finally correlated with the known risk factors in Indian population.

## 2. Materials and Methods

### 2.1. Study Samples

A total of 146 biopsy proven cases in 3 groups, namely, control (*n* = 16), premalignant oral lesions (*n* = 54), and OSCC (*n* = 76), were included in this study. Tissue biopsies and peripheral blood samples were collected from Departments of Oral and Maxillofacial Surgery and Otolaryngology of King George's Medical University, Lucknow, and Department of Oral and Maxillofacial Surgery of Saraswati Dental College, Lucknow. The study was approved by the Institutional Ethics Committee of King George's Medical University, Lucknow, India, in the meeting of March 12, 2011. Written informed consent was obtained from all participants of the study.

Histologically confirmed samples lacking any malignancy or premalignant oral lesions were included as controls in the study. The demographic and clinical history of each patient was recorded on a standard questionnaire. The samples were collected in Trizol reagent (Invitrogen, Carlsbad CA, USA) and stored immediately at −80°C till further processing for DNA and RNA isolation.

### 2.2. Methylation Study

#### 2.2.1. DNA Extraction and Purification

Genomic DNA was isolated by phenol/chloroform extraction method followed by ethanol precipitation as described by Sambrook et al. [33]. DNA was purified using Wizard genomic DNA purification kit (Promega) and after quantification on Picodrop spectrophotometer (Picodrop, UK) at 260/280 nm wavelength it was stored at −20°C till use.

#### 2.2.2. Bisulfite Modification

Bisulfite conversion of genomic DNA (200–500 ng) was performed using commercially available kit (MethylCode Bisulfite conversion kit, Invitrogen, CA). Briefly, DNA from tissue and blood was treated with conversion reagent and incubated in thermal cycler at 64°C for 2.5 hrs, followed by purification and desulphonation steps. Finally, the converted DNA was eluted and stored at −20°C till further use. The bisulfite-treated DNA was used as template for methylation-specific PCR (MSP).

#### 2.2.3. Methylation Specific PCR (MSP)

Methylation was done by MSP method [34] with slight modifications. Nested PCR was performed to analyze the promoter methylation status of the* MGMT* and* p16* genes [35]. During 1st step PCR, the primers recognized the bisulfite modified template but did not discriminate between the methylated and unmethylated alleles. PCR amplification was carried out in a 20 *μ*L reaction mixture containing 10 *μ*L of Platinum Super PCR master mix consisting of Taq DNA polymerase with Platinum Taq Antibody, 22 mM Tris-HCl (pH 8.4), 55 mM KCl, 1.65 mM MgCl_2_, 220 *μ*M of each dNTP (Invitrogen, CA, USA), 1 *μ*L of 10 *μ*M of forward and reverse primers, and 1 *μ*L (250 ng) of bisulfite-treated DNA. The reaction was heated at 95°C for 10 minutes and then amplified for 40 cycles (95°C/30 seconds, 64°C (for p16) or 52°C (for MGMT)/30 seconds, and 72°C/30 seconds), followed by a final 10-minute extension at 72°C. 1st step PCR product was diluted 10-fold and 1 *μ*L was used for the 2nd step PCR, using the same reagents and conditions as in 1st step PCR. Each sample was further amplified in two reactions: one containing primers specific for methylated cytosine and the other containing primers specific for unmethylated cytosine. Each reaction was heated at 95°C for 10 minutes and then amplified for 40 cycles, each consisting (for the reaction containing methylated primers) of 95°C/30 seconds, 70°C (for* p16*) or 64°C (for* MGMT*)/30 seconds, and 72°C/30 seconds, and (for the reaction containing unmethylated primers) of 95°C/30 seconds, 64°C (for both* p16* and* MGMT*)/30 seconds, and 72°C/30 seconds [36]. The nucleotide sequences of the primers for two-stage MSP were described previously by Liu et al. [36]. Methylated and unmethylated genomic DNA (Cells-to-CpG Methylated and Unmethylated genomic DNA Control Kit, Applied Biosystem, CA) were used as positive controls. PCR product was visualized with 4% agarose gel electrophoresis (AGE).

The MSP PCR products were analyzed by restriction fragment length polymorphism (RFLP) to confirm their “methylated” status as described by Liu et al. [36]. Briefly, 2 *μ*L of aliquot from nested MSP product was treated in a final 10 *μ*L reaction with the restriction enzyme Fnu4H1 for the* p16* gene, and with TaqI and BstU1 for the* MGMT* gene using reagents and conditions provided by the manufacturer (New England Biolabs, Beverly, MA). Fnu4H1 and BstU1 restriction enzymes both are methylation sensitive, and TaqI is methylation insensitive. The digested product was separated on 2% AGE and visualized in UV light using gel documentation system. Methylated and unmethylated positive controls DNA were also digested with the same restriction enzymes.

### 2.3. Gene Expression Study

#### 2.3.1. RNA Extraction

RNA was extracted from frozen tissue and blood samples with Trizol reagent (Invitrogen, Carlsbad, CA) [37]. RNA purification was done by DNase1 (Invitrogen, Amplification grade) treatment. In brief, 1 *μ*g of total RNA sample was treated with DNase I (1 U/10 *μ*L) and incubated for 15 min at room temperature followed by enzyme inactivation with 25 mM EDTA at 65°C for 10 min. RNA was quantified on a Picodrop spectrophotometer (Picodrop, UK) at 260/280 nm wavelength.

#### 2.3.2. cDNA Synthesis (Reverse Transcriptase PCR)

250 ng of the total RNA was subjected to reverse transcription PCR using random hexamer primers with high capacity cDNA reverse transcription kit (Applied Biosystems, CA) for cDNA synthesis as per manufacturer's instruction. Briefly, the 20 *μ*L reaction was performed in 3 incubation steps: 25°C for 10 min followed by 37°C for 2 hrs and finally 85°C for 5 min. cDNA was stored at −20°C until use for real-time PCR.

#### 2.3.3. Quantitative Real-Time PCR (qPCR)

qPCR was performed in the presence of SYBR Green fluorescent dye using a StepOne Real-time PCR system (Applied Biosystems, CA, USA). Briefly, the reaction mixture consisted of reverse transcribed cDNA, 2X POWER SYBR Green master mix (Applied Biosystem), and 10 *μ*M of forward and reverse primers. The primer sequences for* MGMT*,* p16*, and **β*-Actin* gene were selected from published articles [38–40] and synthesized by Eurofins MWG Operon, India. Primer sequences were cross-checked by Primer Express software 3.0 (Applied Biosystems, USA) and Blast sequence analysis (National Centre for Biotechnology Information, USA). Relative gene expression was determined by the 2^−ΔΔCt^ method using beta-actin as an endogenous control [37]. A negative control without template was run in parallel to assess the overall specificity of the reaction. All reactions were run in triplicates.

### 2.4. Statistical Analysis

Data was presented as frequencies and percentages for categorical variables. Methylation and unmethylation frequency and their correlation with clinicopathological variables were evaluated by chi-square (*χ* ^2^) test using GraphPad prism software (Version3.0, GraphPad Software, USA). The real-time PCR data was analyzed using Expression Suit software v1.0 (Life Technology, Applied Biosystem). The results were considered statistically significant at a *P* value ≤ 0.05.

## 3. Results

### 3.1. Demographic and Clinical Characteristics

Demographic and clinical characteristics (age, gender, and habits like tobacco, alcohol, bidi, and pan masala consumption) of the patients recruited for the study are summarized in Table 1. Although selection of patients for the study was unbiased, the number of males in the study was more than females. The tobacco consuming habit was more in patients with premalignant oral lesions (90–100%) and OSCC (78%) as compared to controls (56%). Similarly, the number of PMOL and OSCC patients with the habits of alcohol drinking, pan masala chewing and bidi smoking were higher as compared to controls.

### 3.2. Analysis of Gene Promoter Methylation

*MGMT* and* p16* gene showed similar methylation pattern in tissue and blood DNA samples from patients with premalignant oral lesions (leukoplakia with and without dysplasia, SMF, and OLP) and OSCC (Table 2). Significant promoter region methylation of* MGMT* gene was observed in 59% and 76% of tissue samples and 39% and 57% of blood samples in the premalignant oral lesion and OSCC groups, respectively, while 13% methylation was observed in tissue samples and 6% in blood samples of the control group. Significant promoter region methylation of* p16* gene was observed in 57% and 82% of tissue samples and 33% and 70% of blood samples in the premalignant oral lesion and OSCC groups, respectively, while 13% methylation was observed in tissue samples and there was no methylation in blood samples of the controls. We also observed different frequencies of unmethylation in tissue and blood DNA of control, premalignant oral lesions, and OSCC groups; however, the data was not statistically significant. As compared to controls,* MGMT* promoter methylation was significantly higher in tissue and blood samples of premalignant oral lesions and OSCC. Among premalignant oral lesions,* MGMT* methylation frequency in tissue samples was the highest for leukoplakia with dysplasia (73%, *P* = 0.0002) followed by leukoplakia without dysplasia (73%, *P* = 0.0015), SMF (46%, *P* = 0.0437), and OLP (25%, *P* = 0.4386 NS). However, in blood samples methylation was significantly the highest in leukoplakia without dysplasia (55%, *P* = 0.0049) followed by leukoplakia with dysplasia (41%, *P* = 0.0166), SMF (31%, *P* = 0.0821, NS), and OLP (25%, *P* = 0.1904 NS). The* MGMT* gene methylation in the OSCC group was significantly higher than that observed for all subgroups of premalignant oral lesions (*P* = 0.0379 for tissue and 0.0468 for blood samples). PCR products of* MGMT* gene methylation were visualized on 4% AGE.

The* p16* gene methylation frequency in tissue samples of premalignant oral lesions was the highest in leukoplakia with dysplasia (68%, *P* = 0.0007) followed by SMF (62%, *P* = 0.0057), OLP (50%, *P* = 0.0455), and leukoplakia without dysplasia (36%, *P* = 0.1428 NS). In blood samples, this was the highest in SMF (46%, *P* = 0.0023) followed by leukoplakia with dysplasia (45%, *P* = 0.0017) followed by leukoplakia without dysplasia (18%, *P* = 0.0763 NS) and OLP (0%). The methylation for* p16* was significant in tissue and blood samples from all subgroups of premalignant oral lesion and OSCC patients as compared to controls. PCR products of* p16* gene methylation were visualized on 4% AGE. MSP result was confirmed by RFLP. TaqI, BstU1, and FNU4H1 restriction enzymes cut only templates containing methylated cytosines (methylated template). In case of unmethylated templates where the unmethylated cytosines are transformed by bisulfite treatment into uracils (converted to thymidine after PCR), the product is not digested by the enzyme. All methylation positive samples showed presence of digestion products on RFLP (see Supplementary Figures 1(a) and 1(b) in Supplementary Material available online at http://dx.doi.org/10.1155/2014/248419).

### 3.3. Relative Gene Expression of* MGMT* and* p16* Gene

**Figure 1:**
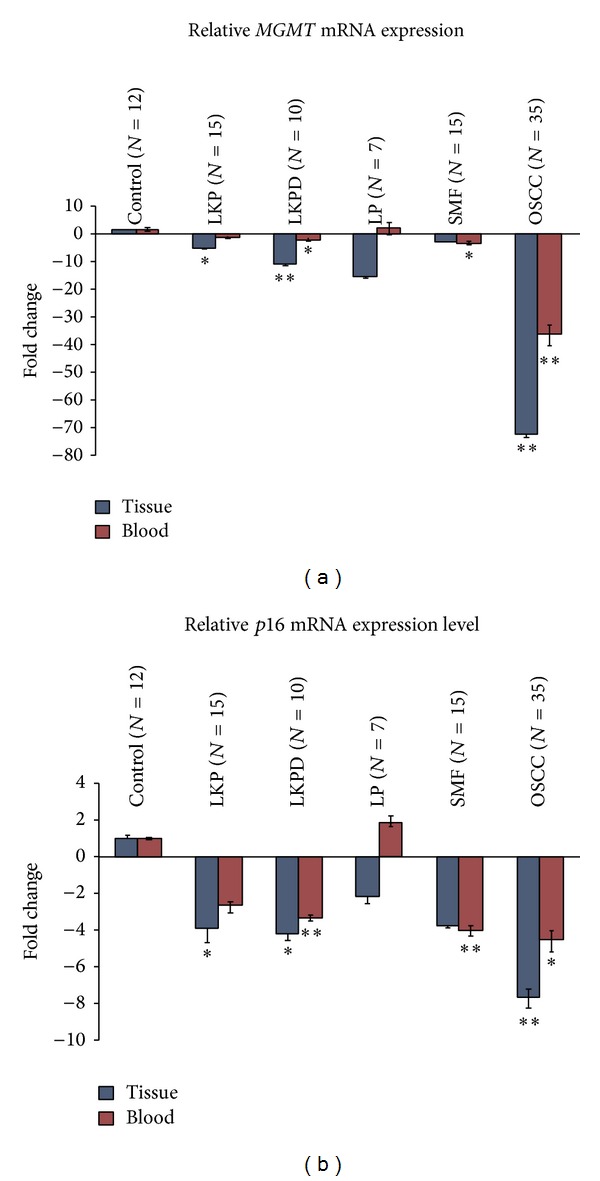
Relative mRNA expression level of* MGMT* and* p16* genes in tissue and blood samples of different groups. Statistically significant values were represented by *P < 0.05, **P < 0.01, and ***P < 0.001.

The mRNA expression of* MGMT* and* p16* genes was significantly downregulated in both tissue and blood samples of premalignant oral lesions (leukoplakia with and without dysplasia, and SMF) and OSCC (*P* ≤ 0.05). Relative* MGMT* and* p16* mRNA expression was nonsignificant both in tissue and blood samples of cases with OLP (Figure 1).

### 3.4. Correlation with Risk Factors

Known risk factors for OSCC, namely tobacco, bidi, alcohol, and pan masala consumption, were found to contribute to promoter region methylation of both* MGMT* and* p16* genes in controls, premalignant oral lesions, and OSCC groups. While tobacco chewing (*P* = 0.041 and *P* = 0.015 for tissue and blood samples, resp.) and bidi smoking (*P* = 0.038 and *P* = 0.048 for tissue and blood samples, resp.) showed significant association with methylation of* MGMT* gene in all groups,* p16* gene methylation was significantly associated with tobacco chewing (*P* = 0.049), pan masala (*P* = 0.007), and bidi smoking (*P* = 0.015) only in tissue samples of all study groups (Table 3).

## 4. Discussion

In the present study, we examined the methylation pattern and gene expression of* MGMT* and* p16* gene in histologically proven biopsies and corresponding blood samples from patients with oral premalignant and malignant lesions and correlated them with known risk factors. We observed significant association of* MGMT* and* p16* gene methylation with oral precancer and cancer.

The methylation frequency was analyzed by MSP, a simple, sensitive, and specific method for determining the promoter hypermethylation status of CpG islands [41]. We observed 76% MGMT methylation frequency in OSCC tissue samples. In DNA from corresponding blood samples of these patients the frequency was 57%. Our results are consistent with those of Kordi-Tamandani et al. who observed 73.7%* MGMT* methylation in tissue samples which was significantly different as compared to controls [42]. In another study, hypermethylation of* MGMT* gene in OSCC was reported to occur in 25–52% of tissue samples [43–45]. Promoter hypermethylation has been reported in premalignant lesions with and without epithelial dysplasia [46, 47]. Liu et al. observed 30.2%* MGMT* methylation in oral leukoplakia [48]. Interestingly, in the present study patients of leukoplakia with and without dysplasia showed promoter methylation in tissue samples of 73% of both cases, respectively. Blood samples of these patients showed 41% and 55% methylation, respectively. We also observed an increased promoter methylation frequency of* MGMT* gene in tissue (46%) and blood samples (31%) from patients with SMF. To the best of our knowledge, there is so far no report available on* MGMT* promoter methylation in blood samples of patients with premalignant oral lesions and OSCC in Indian population. We found significant* MGMT* methylation in blood samples of leukoplakia with and without dysplasia and OSCC groups as compared to controls.

Alterations in* p16* gene are known to affect cell cycle regulation, specifically when suppressing the G1 phase [22]. In the present study,* p16* promoter methylation was observed in tissue samples of 57% cases of premalignant oral lesions and 82% cases OSCC. Shaw et al. observed statistically significant increase in* p16* promoter methylation in patients with oral tumors over normal tissues [45]. In OSCC samples,* p16* methylation has been reported at a frequency of 27–76% [20, 27]. There are available reports which have shown the effect of* p16* methylation in precancerous oral lesions with and without epithelial dysplasia [46, 49] and its association with advanced OSCC [43, 50–52]. von Zeidler et al. evaluated methylation status of* p16* gene in premalignant oral lesions and found 9.7% of cases with promoter hypermethylation [53]. Liu et al. have reported* p16* promoter methylation in 25.6% cases of leukoplakia [48]. In the present study, we found a significant 68% methylation in tissue samples and 45% in blood samples of leukoplakia with dysplasia. Further, the methylation frequency of* p16* in tissue (62%; *P* = 0.0057) and blood (46%; *P* = 0.0023) samples of patients with SMF was statistically significant. Although no studies on* p16* hypermethylation are available in SMF patients, Xu et al. have reported significant hypermethylation of* ECAD* and* COX-2* genes in blood samples from patients with SMF [54]. A recent prospective study also reported that* p16* hypermethylation is significantly increased in precancerous epithelial dysplasia and associated with high rate of OSCC development [55]. These findings confirm that methylation in tumor suppressor genes is an early event that might confer cell growth advantages contributing to the tumorigenic process.

In the present study, both methylated and unmethylated DNA of* MGMT* and* p16* genes were observed in tissue and blood samples of all three study groups. The methylated group included samples showing complete methylation (only methylated amplicon) as well as samples showing partial methylation (both methylated and unmethylated amplicons). The presence of significant level of methylation and unmethylation in DNA from same patient may be a result of cellular heterogeneity of the sample or monoallelic activation [56–58]. Partial methylation observed in our study may be related to a germline first “hit” of an “expanded two hit model,” of epigenetic inactivation suggested by Indovina et al. [59]. Based on this model, the epigenetic first “hit” would inactivate one allele of the both genes in all cells of the body. The partially methylated cells then could acquire a somatic second “hit” (a mutation or a second epigenetic alteration) to progress to cancer [60]. According to this hypothesis, although a methylation level of 50% was expected in peripheral blood samples of retinoblastoma patients, deviations from this value were observed in most of the reported cases [59]. Pertinently, in the present study, we observed* MGMT* promoter methylation in 39% and 57% blood samples of patients with premalignant oral lesions and OSCC, respectively. Significant* p16* promoter methylation was observed in 33% and 70% blood samples of patients with premalignant oral lesions and OSCC, respectively.

Tumors that have metastasized may not shed many cells into the peripheral blood but might release tumor DNA into the circulation. Based on this finding, detection of genetic alterations and methylation abnormalities in the blood sample of cancer patients may thus have a profound impact on noninvasive diagnosis of cancers among high-risk populations. We screened blood samples of the biopsy-proven cases and controls and observed that the methylation frequency of both the genes followed the same pattern as in tissue samples of patients with premalignant oral lesions and OSCC. Methylation frequency in premalignant oral lesions and OSCC was found to be significantly higher than controls suggesting use of peripheral blood as noninvasive source for early detection of oral cancer and as a surrogate marker to detect epigenetic changes. Promoter hypermethylation of* MGMT* and* p16* gene in peripheral blood has been reported in primary lung cancer and nonsmall cell lung cancer patients [15, 61].

The functional consequence of promoter hypermethylation is transcriptional silencing of the associated genes [62]. Promoter region hypermethylation-induced* MGMT* gene silencing has been reported in glioblastoma multiforme, head, and neck squamous cell carcinomas in general, and also specifically in oral cavity cancer [17, 42, 63, 64]. Significant downregulation of* p16* gene expression due to promoter hypermethylation has been reported in colorectal cancer and oral cancer [65, 66]. In order to confirm the effect of promoter methylation on gene expression in our cases, we analyzed methylation status and mRNA levels of both* MGMT* and* p16* genes in tissue and blood samples from various study groups. Significant downregulation of* MGMT* and* p16* gene expression was observed in both tissue and blood samples from premalignant oral lesions and OSCC patients. It is known that downregulation of* MGMT* gene results in decreased formation of AGT protein which repairs potentially mutagenic and cytotoxic alkylation of DNA by removing adducts from the O^6^ position on guanine [16]. The silencing of* p16* gene expression leads to decreased expression of* p16* protein which plays an important role as a direct inhibitor of cyclin-dependent kinases (CDK4 and CDK6) to exert its oncosuppressor activity. Decrease or loss of* p16* protein expression in the nuclei of tumor cells has also been reported in many kinds of human carcinomas [67].

In India, the main risk factors for premalignant oral lesions and OSCC are tobacco, bidi, pan masala (betel quid), alcohol consumption, and unhygienic conditions with poor nourishment [68, 69]. High promoter methylation frequency of* MGMT* and* p16* genes was observed in patients with premalignant oral lesions and OSCC who were tobacco chewers and bidi smokers indicating contribution of these risk factors to gene silencing by promoter hypermethylation. Our findings are in agreement with previous studies that have shown an association between tobacco consumption in chewed or smoked form and hypermethylation of both the genes in HNSCC [51, 53, 70].

The strength of our study is the well-characterized population of premalignant oral lesions and OSCC, which has detailed baseline epidemiological data on risk factors. In addition, a comparison between tissue and peripheral blood methylation from the same patients was possible because of the availability of both biological samples. Possible limitations of this study are that although this includes different subtypes of premalignant oral lesions and comparison between tissue and blood, the sample size is relatively small and longitudinal data could not be provided due to lack of follow-up data of patients with premalignant oral lesions.

To summarize, hypermethylation-induced transcriptional silencing of* MGMT* and* p16* genes in both oral precancer and cancer distinctly separate from normal oral mucosa suggests an important role of these changes in progression of premalignant state to malignancy. Detection and quantitation of promoter region methylation may provide lesion-specific epigenetic profile and contribute significantly to screening, surveillance, and management of premalignant oral lesions and OSCC. Aberrant methylation of* MGMT* and* p16* promoter could thus be a useful adjunct to histopathologic evaluation for prediction of risk of malignant transformation of a precancerous state. The altered DNA methylation site of the cancer epigenome is not limited to promoter hypermethylation of select genes but also includes global hypomethylation as a prelude to tumor suppressor gene inactivation, oncogene activation, and genome instability. However, an obvious limitation of the present study is that it is a cross-sectional study.

Long-term follow-up studies including measurement of DNA methylation status in promoter and nonpromoter regions (global) are required to determine the functional relevance of these alterations. Further, similar pattern of methylation for both* MGMT* and* p16* genes in tissue and blood samples of premalignant oral lesions and OSCC suggests use of blood as a potential substitute to tissue samples for screening or diagnosing precancer and early malignancy.

## Supplementary Material

MSP products of *MGMT* and *p16* genes were digested by restriction endonucleases viz. TaqI, BstU1, and FNU4H1 (supplementary fig 1a and 1b). All methylation positive samples showed presence of digestion products.

## Figures and Tables

**Table 1:** Mean age and sex of patients and their distribution according to exposure to risk factors.

Features	Control (*n* = 16)	PMOLs (*n* = 54)				OSCC (*n* = 76)
		LKP (*n* = 22)	LKPD (*n* = 11)	SMF (*n* = 13)	OLP (*n* = 8)	
Age ± SD (Yrs)	29 ± 8.28	39 ± 13.59	34 ± 11	40 ± 13	34 ± 8	53 ± 12.97
Sex (M/F)	12/4	19/3	10/1	11/2	7/1	69/7
Tobacco consumers	9 (56%)	20 (91%)	10 (91%)	13 (100%)	8 (100%)	59 (78%)
Alcohol consumers	5 (31%)	6 (27%)	3 (27%)	—^#^	3 (38%)	19 (25%)
Pan masala users	3 (19%)	10 (45%)	7 (64%)	3 (23%)	5 (63%)	23 (30%)
Bidi smokers	10 (62.5%)	15 (68%)	7 (64%)	8 (62%)	5 (62.5%)	43 (57%)

M: male, F: female; SD: standard deviation. PMOLs: premalignant oral lesions; LKP and LKPD: leukoplakia with and without dysplasia; SMF: submucosal fibrosis; OLP: oral lichen planus.

^#^Out of 13 SMF patients none of the patients was found to have the habit of alcohol drinking.

**Table 2:** Methylation profile of *MGMT* and *p16* genes in tissue and blood samples of study groups.

Gene methylation	Groups	Tissue			Blood		
		M*	U	*P* value^a^	M*	U	*P* value^a^
*MGMT *	Control (*n* = 16)	2 (13%)	14 (87%)	**Ref**	1 (6%)	15 (94%)	**Ref**
	PMOLs (*n* = 54)	32 (59%)	22 (41%)	**0.001**	21 (39%)	33 (61%)	**0.0135**
	PMOL subtypes						
	LKP (*n* = 22)	16 (73%)	6 (27%)	**0.0002**	9 (41%)	13 (59%)	**0.0166**
	LKPD (*n* = 11)	8 (73%)	3 (27%)	**0.0015**	6 (55%)	5 (45%)	**0.0049**
	SMF (*n* = 13)	6 (46%)	7 (54%)	**0.0437**	4 (31%)	9 (69%)	0.0821
	OLP (*n* = 8)	2 (25%)	6 (75%)	0.4386	2 (25%)	6 (75%)	0.1904
	OSCC (*n* = 76)	58 (76%)	18 (24%)	**0.0001**	43 (57%)	33 (43%)	**0.0002**

*P* value^b^	PMOLs versus OSCC	—	—	**0.0379**	—	—	**0.0468**

*p16 *	Control (*n* = 16)	2 (13%)	14 (87%)	**Ref**	0 (0%)	16 (100%)	**Ref**
	PMOLs (*n* = 54)	31 (57%)	23 (43%)	**0.0016**	18 (33%)	36 (67%)	**0.0074**
	PMOL subtypes						
	LKP (*n* = 22)	15 (68%)	7 (32%)	**0.0007**	10 (45%)	12 (55%)	**0.0017**
	LKPD (*n* = 11)	4 (36%)	7 (64%)	0.1428	2 (18%)	9 (82%)	0.0763
	SMF (*n* = 13)	8 (62%)	5 (38%)	**0.0057**	6 (46%)	7 (54%)	**0.0023**
	OLP (*n* = 8)	4 (50%)	4 (50%)	**0.0455**	0 (0%)	8 (100%)	NA
	OSCC (*n* = 76)	62 (82%)	14 (18%)	**0.0001**	53 (70%)	23 (30%)	**0.0001**

*P* value^b^	PMOLs versus OSCC	—	—	**0.0026**	—	—	**0.0001**

M: methylated, U: umethylated, and Ref: reference; ^a^Chi-square test considering control as reference category; ^b^Chi-square test considering PMOLs as reference category.

*Methylated group includes samples showing complete methylation (only methylated amplicon) as well as samples showing partial methylation (both methylated and unmethylated amplicons).

**Table 3:** Correlation of promoter methylation with risk factors in different groups.

Genes	Risk factors	Methylated	Control (*n* = 16)	PMOLs (*n* = 54)	OSCC (*n* = 76)	*P* value^a^
*MGMT *	Tobacco chewers (*N*)	*N *	9	51	59	
		Tissue	2 (22%)	24 (47%)	52 (88%)	**0.041**
		Blood	1 (11%)	14 (27%)	39 (66%)	**0.015**
	Alcohol consumers (*N*)	*N *	5	12	19	
		Tissue	0 (0%)	8 (67%)	10 (53%)	0.1588
		Blood	0 (0%)	7 (58%)	7 (37%)	0.259
	Pan masala (*N*)	*N *	3	25	23	
		Tissue	1 (33%)	15 (60%)	14 (61%)	0.877
		Blood	0 (0%)	8 (32%)	14 (61%)	0.236
	Bidi smokers (*N*)	*N *	10	35	43	
		Tissue	2 (20%)	20 (57%)	38 (88%)	**0.038**
		Blood	1 (10%)	15 (43%)	33 (77%)	**0.048**

*p16 *	Tobacco chewers (*N*)	*N *	9	51	59	
		Tissue	2 (22%)	25 (49%)	51 (86%)	**0.049**
		Blood	0 (0%)	18 (35%)	50 (85%)	0.088
	Alcohol consumers (*N*)	*N *	5	12	19	
		Tissue	0 (0%)	8 (67%)	11 (58%)	0.124
		Blood	0 (0%)	6 (50%)	8 (42%)	0.278
	Pan masala (*N*)	*N *	3	25	23	
		Tissue	2 (67%)	19 (76%)	18 (78%)	**0.015**
		Blood	0 (0%)	11 (44%)	17 (74%)	0.107
	Bidi smokers (*N*)	*N *	10	35	43	
		Tissue	2 (20%)	29 (83%)	40 (93%)	**0.007**
		Blood	0 (0%)	15 (43%)	38 (88%)	0.071

^a^Chi-square test; *n* = total number of samples in the group; *N* = number of individuals exposed to risk factors in different groups. Significant *P* value represented with bold.
