# Supplementary material for: Promoter Region Hypermethylation and mRNA Expression of MGMT and p16 Genes in Tissue and Blood Samples of Human Premalignant Oral Lesions and Oral Squamous Cell Carcinoma
Source: Biomed Res Int. 2014 Jun 2;2014:248419. doi: 10.1155/2014/248419 (PMC4058681; doi:10.1155/2014/248419)
Supplement: Supplementary file 1 — MSP products of MGMT and p16 genes were digested by restriction endonucleases viz. TaqI, BstU1, and FNU4H1 (supplementary fig 1a and 1b). All methylation positive samples showed presence of digestion products. [file 248419.f1.pdf]

Supplementary figure 1a: Restriction endonuclease digestion of methylated and unmethylated PCR product of *MGMT* gene

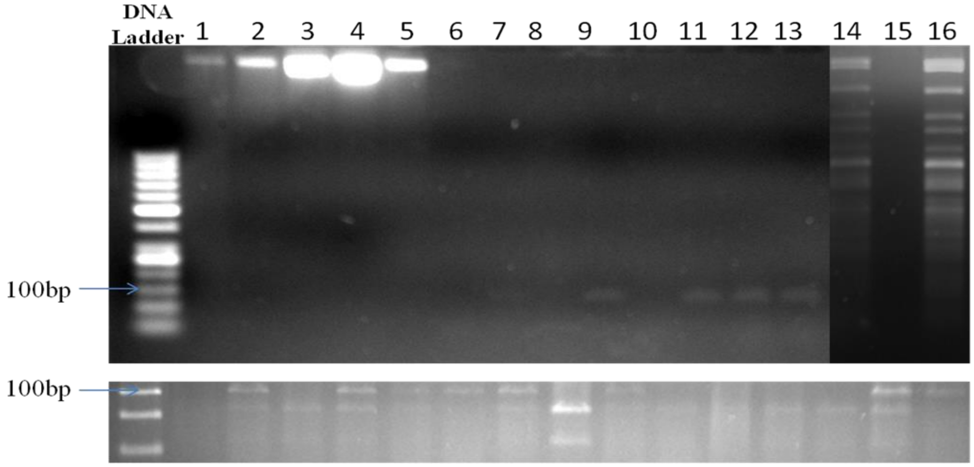

Gel 1: Well 1 to 3: Unmethylated control DNA treated with BstU1; 4, 5: Methylated control DNA treated with Taq1; 6 to 13: methylated and unmethylated PCR products (without treated with any enzyme); 14, 16: methylated gDNA treated with BstU1; well 15: no sample. Gel 2: Methylated positive digested with BstU1. DNA ladder: 25bp

Supplementary figure 1b: Restriction endonucleases digestion of methylated and unmethylated PCR product of *p16* gene

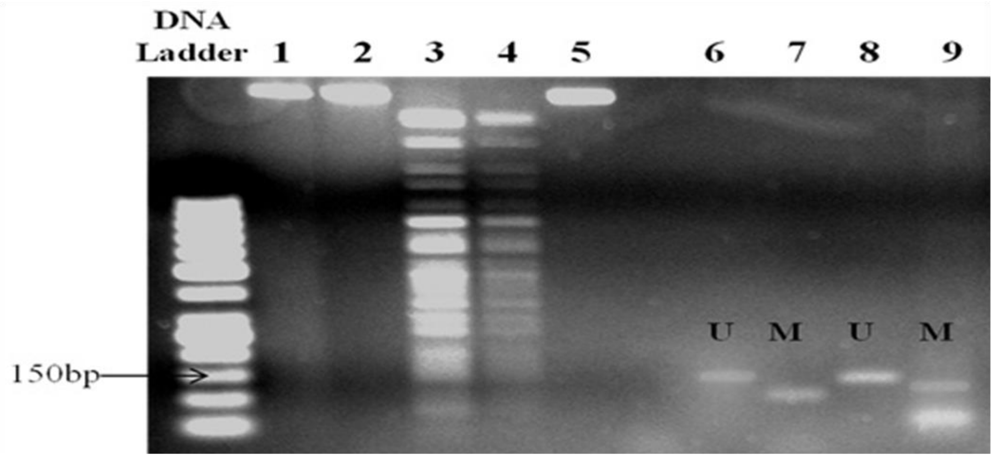

Well 1, 2 and 5: Unmethylated control DNA; 3, 4: Methylated control DNA; 6, 7: MSP PCR product: not treated with enzyme; 8, 9: PCR product with enzyme treatment. U: unmethylated (151bp); M: Methylated (150bp). DNA ladder: 50bp
